# Semi-automatic segmentation of elongated interventional instruments for online calibration of C-arm imaging system

**DOI:** 10.1007/s11548-025-03434-w

**Published:** 2025-06-26

**Authors:** Negar Chabi, Alfredo Illanes, Oliver Beuing, Daniel Behme, Bernhard Preim, Sylvia Saalfeld

**Affiliations:** 1 Faculty of Computer Science, Otto-von-Guericke University, Universitätsplatz 2, 39106 Magdeburg, Germany; 2 Department of Radiology, AMEOS Hospital Bernburg, Kustrenaer Str. 98, 06406 Bernburg, Germany; 3 Clinic for Neuroradiology, University Hospital of Magdeburg, 39120 Magdeburg, Germany; 4 Forschungscampus STIMULATE, Magdeburg, Germany; 5 University Hospital Schleswig-Holstein Campus Kiel, 24118 Kiel, Germany

**Keywords:** Online-calibration, Biplane X-ray imaging system, Perspective projection, Digital subtraction angiography, C-arm, Segmentation, Catheter, Machine learning

## Abstract

**Purpose:**

The C-arm biplane imaging system, designed for cerebral angiography, detects pathologies like aneurysms using dual rotating detectors for high-precision, real-time vascular imaging. However, accuracy can be affected by source-detector trajectory deviations caused by gravitational artifacts and mechanical instabilities. This study addresses calibration challenges and suggests leveraging interventional devices with radio-opaque markers to optimize C-arm geometry.

**Methods:**

We propose an online calibration method using image-specific features derived from interventional devices like guidewires and catheters (In the remainder of this paper, the term”catheter” will refer to both catheter and guidewire). The process begins with gantry-recorded data, refined through iterative nonlinear optimization. A machine learning approach detects and segments elongated devices by identifying candidates via thresholding on a weighted sum of curvature, derivative, and high-frequency indicators. An ensemble classifier segments these regions, followed by post-processing to remove false positives, integrating vessel maps, manual correction and identification markers. An interpolation step filling gaps along the catheter.

**Results:**

Among the optimized ensemble classifiers, the one trained on the first frames achieved the best performance, with a specificity of 99*.*43% and precision of 86*.*41%. The calibration method was evaluated on three clinical datasets and four phantom angiogram pairs, reducing the mean backprojection error from 4*.*11 ± 2*.*61 to 0*.*15 ± 0*.*01 mm. Additionally, 3D accuracy analysis showed an average root mean square error of 3*.*47% relative to the true marker distance.

**Conclusions:**

This study explores using interventional tools with radio-opaque markers for C-arm self-calibration. The proposed method significantly reduces 2D backprojection error and 3D RMSE, enabling accurate 3D vascular reconstruction.

## Introduction

Minimally invasive neuroradiological interventions treat cerebrovascular pathologies, like aneurysms, using catheters under fluoroscopic guidance. Low signal-to-noise ratios in low-dose images and overlapping structures hinder catheter visualization [1]. Ambrosini et al. [2] proposed a U-Net-based deep network for real-time catheter segmentation in 2D X-ray fluoroscopy, but it showed false negatives and segmentation gaps, highlighting the need for larger datasets. Similarly, Chen et al. [3] achieved high localization accuracy with deep learning, though such methods risk overfitting without sufficient labeled data. Automating catheter segmentation and tracking can enhance image guidance in endovascular procedures.

Biplane angiography captures high-resolution 2D vessel images but lacks 3D vascular detail, complicating the analysis of complex pathologies like AVMs [4]. 3D-DSA provides clearer angiographic details and, in 89% of cases, offers improved anatomical insights, with 43% showing superior visualization compared to 2D-DSA [5]. Although 3D reconstruction from rotational DSA or tomographic imaging methods like CTA and MRA addresses this limitation, these methods struggle to resolve vessels smaller than 1 mm and often lack flow information [4, 6].

3D rotational DSA, though similar to 2D-DSA, involves multiple projections, resulting in higher radiation doses that primarily affect the patient. Reconstructing 3D vessel models from 2D-DSA images is crucial for planning and monitoring complex brain surgeries, enabling better visualization of feeding, draining, and nearby vessels to minimize complications like hemorrhage [4]. Accurate vascular reconstruction requires precise knowledge of the system’s geometry, including rotation and translation parameters between projection views for each C-arm configuration [7].

Although angiographic parameters are recorded in DICOM headers, projection matrices derived from them may be inaccurate due to intrinsic parameter uncertainty, table movement, imprecise determination of orientation from recorded parameters, and mismatched isocenters [8].

Online calibration can utilize bifurcation points [9], but these are limited in cases like carotid or vertebral vasculature or acute proximal total coronary obstructions [10]. Although calibration phantoms can reliably determine the exact system geometry, they are often impractical in intervention settings as they may interfere with the already complex clinical environment. [9, 11]. Elongated interventional tools with radio-opaque markers simplify correspondence identification across views. Vachon et al. [12] proposed an online calibration algorithm for 3D pulmonary artery reconstruction using catheters or guidewires as reference structures. Their approach aimed to enhance 3D imaging in interventional procedures. However, the study did not provide detailed validation results for the reconstructed models or specify the method used for selecting corresponding points. This limitation is particularly relevant in cases involving multiple intersections within highly curved vascular structures, where accurate correspondence selection is crucial for reliable reconstruction.

In our previous work [7], we introduced a semi-automatic C-arm self-calibration technique that relied on radio-opaque markers attached to interventional instruments as corresponding points. However, this method faced challenges when there were insufficient markers, leading to potential inaccuracies in the calibration process. In this work, we extend our previous method to address these limitations and further improve the robustness of the calibration process.

We propose using interventional tools with radio-opaque markers for C-arm online calibration and introduce a catheter segmentation method for neuroradiology. A two-stage semi-automatic machine learning algorithm is proposed for catheter detection and segmentation, followed by iterative nonlinear optimization to minimize a cost function (Eq. 6) that accounts for backprojection errors and directional vector discrepancies between actual and the backprojected reconstructed catheter centerlines.

## Materials and methods

### Data acquisition for segmentation

Interventional X-ray angiographies (Artis Q, Siemens Healthineers) were retrospectively analyzed. The training set includes 18 first-frame images and multiple frames (168 images) across 7 datasets, while the test set comprises 13 sequences from 6 clinical datasets. All images have a pixel size of 0.154 mm.

**Fig. 1:**
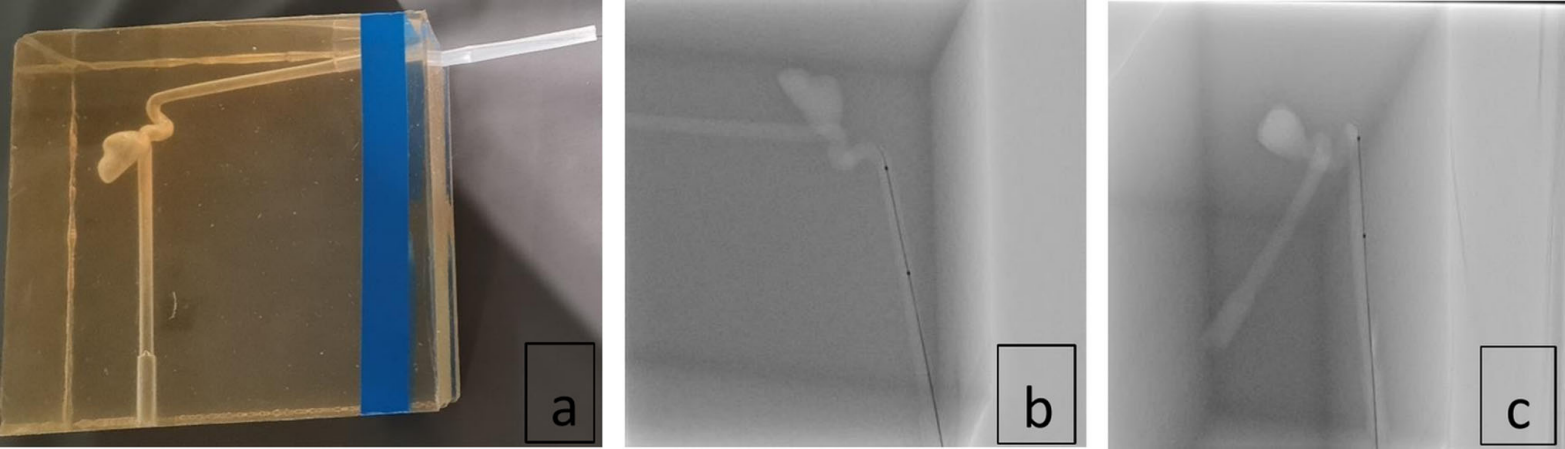
**a** A silicon phantom of an aneurysm-like structure; Biplane views of a silicon phantom with a marked microcatheter inside. **b** 1st viewing angle: *RAO*\*LAO* =  − 14*.*60^◦^*,CAUD*\*CRAN* =  − 30*.*30^◦^, **c** 2nd viewing angle: *RAO*\*LAO* = 73*.*70^◦^*,CAUD*\*CRAN* =  − 26*.*20

Furthermore, to validate the method in 3D, a microcatheter with two radio-opaque markers (30 mm apart) was placed in a vascular silicone phantom with an aneurysm-like structure (Fig. 1).

### Elongated interventional tools segmentation

**Fig. 2:**
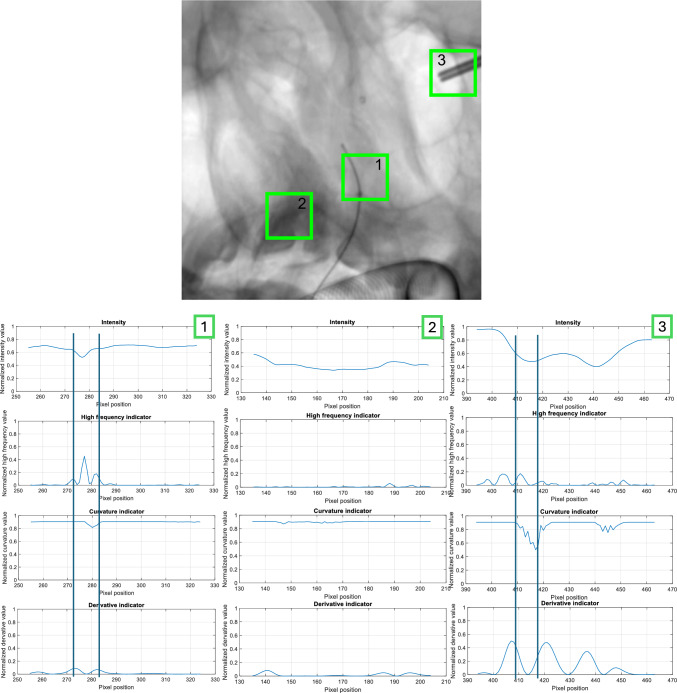
Intensity profile, high-frequency, curvature, and derivative indicators for three ROIs: (1) with catheter, and (2 & 3) without catheter

**Fig. 3:**
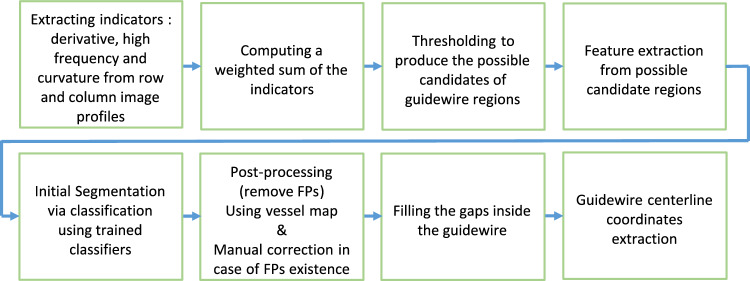
Elongated interventional instruments segmentation workflow

We propose a multi-step approach for catheter detection and segmentation in angiograms, where the catheter appears as a sharp, negative deflection with rapid, peaky changes (Figs. 2 and 3), indicating that catheter events (pixels containing the catheter) should exhibit high derivative and high-frequency characteristics. Horizontal and vertical lines are extracted to evaluate three indicators: high frequency, derivative, and curvature. Figure 2 shows the behavior of these indicators in three regions of an angiogram: one with the catheter and two without, with the third region containing a low-intensity object.

#### Catheter candidate regions computation

The high-frequency signal is derived by squaring the sum of the first three detail coefficients from a ‘db4’ wavelet decomposition. The signal’s trend along image rows and columns is extracted from the approximation part using the discrete wavelet transform (DWT) with Daubechies 4. This trend is subtracted from the raw signal to obtain deviations [13]. The curvature indicator is computed from the negative parts of the subtracted signal (representing catheter regions) using the method in [14], with a 2nd-degree polynomial fitted within a sliding window of seven pixels to highlight catheter peaks. Additionally, a differentiation filter is applied to the subtracted signal to measure the slope of intensity changes, yielding the derivative indicator [15, 16]. Indicators are processed using a 5-pixel sliding window to localize features.

Within each window, the maximum high-frequency value and the mean derivative/curvature values were combined using empirically selected weights ([0.8, 0.1, 0.1], [0.33, 0.33, 0.34], [0.5, 0.25, 0.25], and [0.65, 0.17, 0.18]). Nine combinations were tested by either multiplying the weighted matrix with the high-frequency matrix or directly using the weighted matrix. Otsu’s thresholding is applied to identify catheter regions, and results are compared to a manually annotated ground truth mask created by the author.

Performance metrics (accuracy, sensitivity, specificity, FDR, and precision) show that the [0.8, 0.1, 0.1] weights with multiplication perform best.

An empirically selected threshold of 0.1 prioritizes catheter regions while minimizing false negatives. Probable regions are detected, and a 5-pixel neighborhood is chosen horizontally and vertically, as catheter widths in the dataset do not exceed five pixels.

### Features computation

A total of 64 features were analyzed. The first nine capture the maximum, minimum, and mean values of the squared high-frequency signal, derivative, and curvature, with another nine derived after smoothing with a 5-pixel sliding window (Table 1). Ten additional features represent signal-based statistical metrics (features 19–28) [17].

**Table 1:** Summary of extracted signal-based statistical features

Feature (No.)	Equation	Extracted metrics
Squared high frequency and smoothed version (1–6)	$hf\left( t \right) = \left( {\sum \, D\_j\left( t \right)} \right)^{2}$ $D\_j\left( t \right) = I{\text{DWT}}\left( {C,L,{\text{type}},j} \right)$ $\left[ {C,L} \right] = {\text{DWT}}\left( {{\text{sig}},{\text{level}},{\text{type}}} \right)$	Maximum, Minimum, and Mean
Derivative and smoothed version (7–12)	${\text{der}}\left( k \right) = \left( {y\left( {k + 6} \right)} \right)^{2}$ $y\left( k \right) = \Sigma B\_m \cdot x\_\det \left( {k - m} \right) - \Sigma \, A\_n \cdot y\left( {k - n} \right)$ $A = \left[ {A_{1} ,..., \, A\_N} \right],\;B = \left[ {B_{0} ,...,B\_M} \right]$^1^	Maximum, Minimum, and Mean
Curvature and smoothed version (13–18)	${\text{cur}}\left( k \right) = \theta_{2} \left( k \right) \cdot \theta_{0} \left( k \right)$ $\left[ {\theta_{2} \left( k \right),\;\theta_{0} \left( k \right)} \right] = \left( {X^{T} WX} \right)^{ - 1} \cdot \le X^{T} Wy,$^2^ $X = \left[ { - w^{2} ,1} \right],\;W = {\text{diag}}\left( {d\_sw} \right)^{{}}$ $d\_sw = \left[ {{\text{weights}},\;{1},\;{\text{weights}}} \right]$ ${\text{weights}} = 1:\left( { - 1/h} \right):\left( {1/h} \right)$	Maximum, Minimum, and Mean
Statistical Metrics (19–28)	${\text{RMS}}\left( x \right) = {\text{sqrt}}\left( {\left( {1/N} \right)\Sigma \times \left( k \right)^{2} } \right),\;N = {\text{total}}\;{\text{samples}}$	RMS
	$k = E[(x - \mu )^{4} ]/\sigma^{4}$ $\mu :{\text{mean}},\;\sigma :{\text{std}}\;{\text{dev}}$	Kurtosis
	$s = E[(x - \mu )^{3} ]/\sigma^{3}$ $\mu :{\text{mean}},\;\sigma :{\text{std}}\;{\text{dev}}$	Skewness
	$IF = \frac{{x}_{p}}{\frac{1}{N}\sum_{i = 1}^{N}\left|{x}_{i}\right|}$	Impulse Factor
	$\text{CF}=\frac{{\text{x}}_{\text{p}}}{{\left(\frac{1}{\text{N}}{\sum }_{\text{i}=1}^{\text{N}}\sqrt{{\text{x}}_{\text{i}}}\right)}^{2}}$	Clearance Factor
	${\text{CrestF}} = \frac{{{\text{x}}_{{\text{p}}} }}{{\sqrt {\frac{1}{{\text{N}}}\mathop \sum \nolimits_{{{\text{i}} = 1}}^{{\text{N}}} {\text{x}}_{{\text{i}}}^{2} } }}$	Crest Factor
	$\upsigma =\sqrt{\frac{1}{\text{N}}{\sum }_{\text{i}=1}^{\text{N}}{\left({\text{x}}_{\text{i}}-\upmu \right)}^{2}}$	Standard Deviation
	$\text{log}\left({\text{sigma}}^{2}\right)$	Log Variance
	${\text{sum}}_{\text{i}=1}^{\text{N}-1}\left|{\text{x}}_{\text{i}+1}-{\text{x}}_{\text{i}}\right|$	Variation Metric_1
	$\left|{\sum }_{\text{i}=1}^{\text{N}-1}\left({\text{x}}_{\text{i}+1}-{\text{x}}_{\text{i}}\right)\right|$	Variation Metric_2
Frangi (29–31)	$\text{Frangifilter}\left(\text{x}\right)$ [19]	Maximum, Minimum, and Mean
Difference of Gaussian (32–37)	$\text{DoG }=\text{ normalize}(( {\text{G}}_{{\upsigma }_{1}}*\text{I}) - ( {\text{G}}_{{\upsigma }_{2}}*\text{I }))$ ^3^	Maximum, Minimum, and Mean
Laplacian of Gaussian (38–43)	$\text{LoG}=\text{normalize}\left({\text{LoG}}_{\upsigma }* I\right)$ ^4^	Maximum, Minimum, and Mean
Bottom-hat (44–46)	$\text{bottomhat}\left(\text{x}\right)$ [21]	Maximum, Minimum, and Mean
Directional Information (47–64)	The Hough transform is applied to an edge-detected image, with edge detection performed using the Sobel filter	Refer to text in 2.3 and Fig. 4

^1^The filter coefficients A and B define the recursive and non-recursive components of the filtering operation

^2^*y* is the windowed segment of the signal

^3^ ${\text{G}}_{\upsigma }$ is a Gaussian filter with standard deviation $\upsigma$, '*' is the convolution operator, ${\sigma }_{1}=1$ and ${\upsigma }_{2}=2$, normalize(·) maps the result to the range [0, 1]

^4^ ${\text{LoG}}_{\sigma }$ is the Laplacian of Gaussian (LoG) filter with size 15 and standard deviation$\upsigma = 0.5$

Features 29–31 are derived from the Frangi vesselness filter [18], which assesses vessel-like regions using parameters: scale range [1 2], scale ratio 0.5, and *β*_2_ = 30. For each event, the maximum, mean, and minimum Frangi values are calculated. The implementation used was provided by [19].

Features 32–43 (Table 1) are based on edge detection using Difference of Gaussian (DoG) and Laplacian of Gaussian (LoG) methods [20]. The DoG filter uses Gaussian kernel sizes and standard deviations of 10, 20, 1, and 2, while the LoG filter uses a kernel size of 15. Both are applied to the original and bottom-hat-filtered images, extracting the average, maximum, and minimum of event regions.

Features 44–46 (Table 1) utilize a bottom-hat filter with a disk-shaped structural element (radius three) to remove the background and preserve small dark objects, extracting statistical features, such as the average, maximum, and minimum of event regions.

**Fig. 4:**
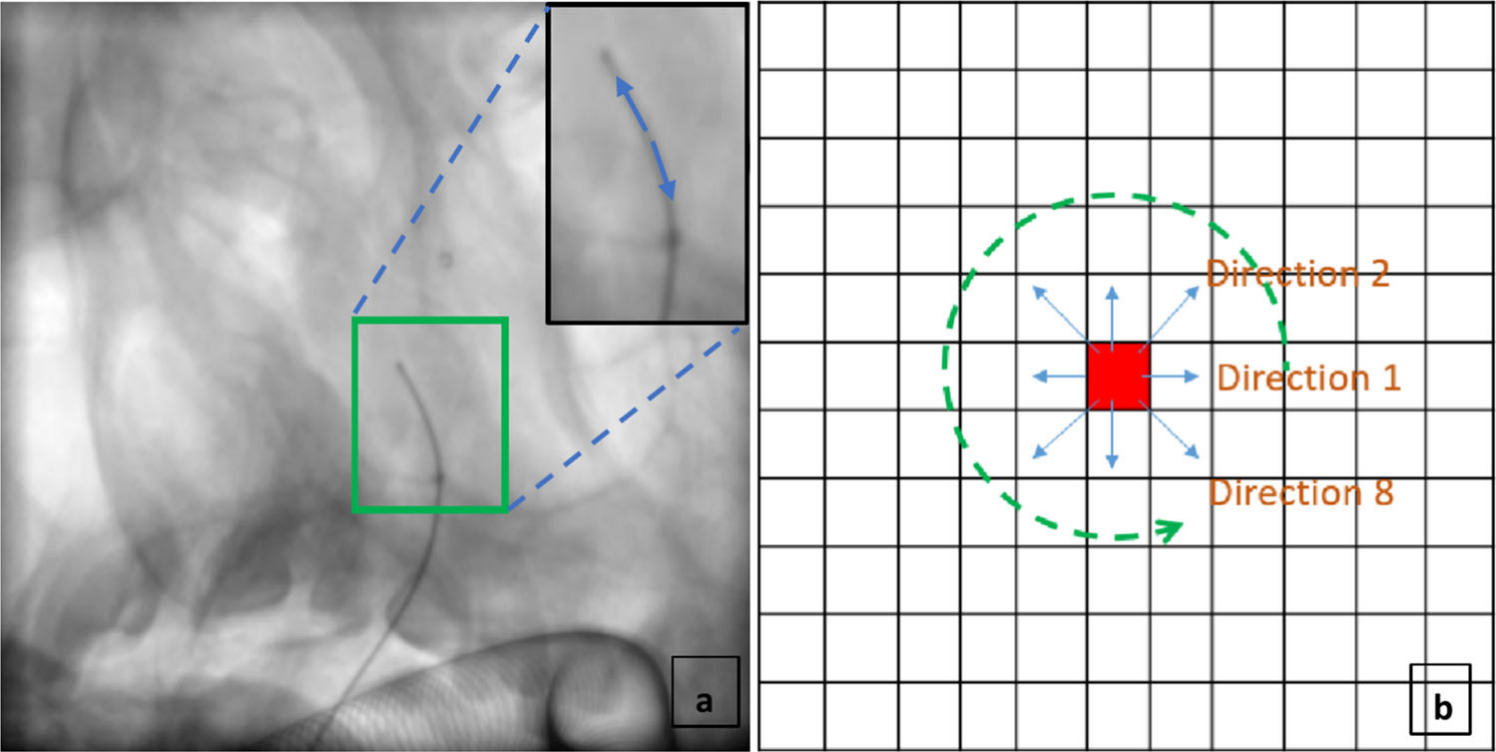
Directional information computation along curvature-like structure; **a** The probability of being catheter is higher along the catheter path, **b** Directional information computed on the curve detection results

Figure 4 shows that catheter presence is more likely along its direction (blue arrows). The Hough transform, typically used for line detection, is applied to capture directional information of curvature-like structures by approximating curves as lines in small scales (e.g., within 11 × 11 pixel patches). The result is a binary image includes probable curves. Values along eight directions within patches are summed for both the binary curvature image and the high-frequency values within the curves. In total, 18 features are extracted, including the directional sums and the total across all directions.

### Learning conditions

Features from catheter candidate regions (first and multiple frames) were used to train classifiers. The training set included 18 first-frame images and 168 multi-frame images from 7 datasets, while the test set comprised 13 sequences from 6 clinical datasets. A 25% holdout validation assessed model performance. MATLAB’s Classification Learner App optimized hyperparameters by minimizing classification errors through automated search.

Tested classifiers included Ensembles, KNN, and SVM, with and without hyperparameter optimization. Ensemble models used Bayesian optimization with GentleBoost, 480 learners, and a learning rate of 0.006.

### Post-processing

**Fig. 5:**
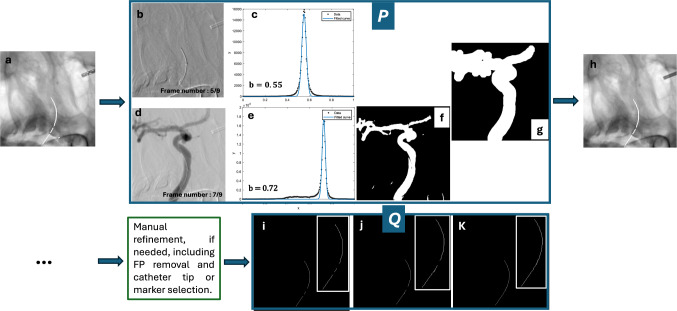
Post-processing; **a** Catheter candidates; P block. Creating vessel mask; **b** (*b*: 0*.*55) & **d** (*b*: 0*.*72) frame discarded and considered for vessel mask creation respectively, **f** Vessel extracted for”d” using kmeans clustering, **g** Final vessel mask, obtained by summing vessel masks over eligible frames followed by smoothing, gap filling, and dilation. **h** Post-processed region, discarding regions outside the vessel mask. Q block. Catheter centerline extraction & Gap filling using AKIMA (Iterative process, **k** Final catheter centerline)

X-ray angiography sequences, enhanced by contrast agents, highlight vessels for creating a vessel map to eliminate false positives (FPs), as instruments are always within vessels. Digital subtraction was applied to middle frames using the first frame as reference [22]. DSA histograms were fitted with a Gaussian function, and frames with peak centers (*b* < 0*.*65) were discarded. K-means clustering [23] separated vessel and non-vessel pixels. Binarized frames were summed into a vessel map, refined through smoothing, gap filling, and dilation. The largest connected region was retained, and FPs outside this map were removed (Fig. 5).

A user was prompted to select two specific points on the catheter (e.g., the catheter tip and an attached opaque marker) in both views. The length of the catheter defined between these points was then used for calibration.

A gap-filling algorithm ensured continuity in catheter skeletons after segmentation. Morphological operations were used to remove spurs, fill regions, and generate a binary skeleton. Disconnected components were linked by interpolating between endpoints using Akima cubic Hermite interpolation, producing a continuous skeleton (Fig. 5) [24].

### Self-calibration of a biplane X-ray imaging system

**Fig. 6:**
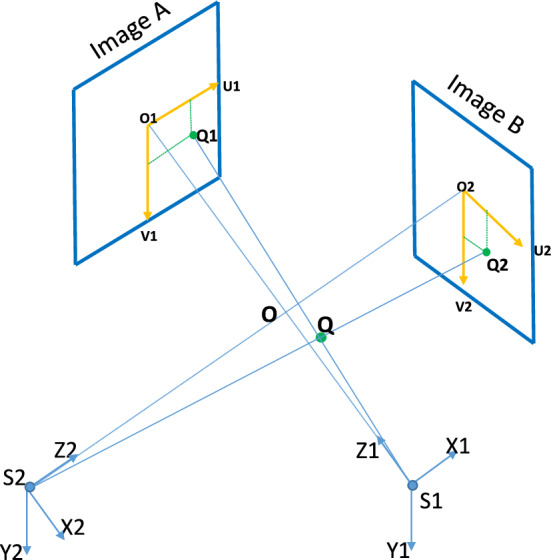
Coordinate systems used to describe the geometry of a biplane angiographic system [27]

**Fig. 7:**
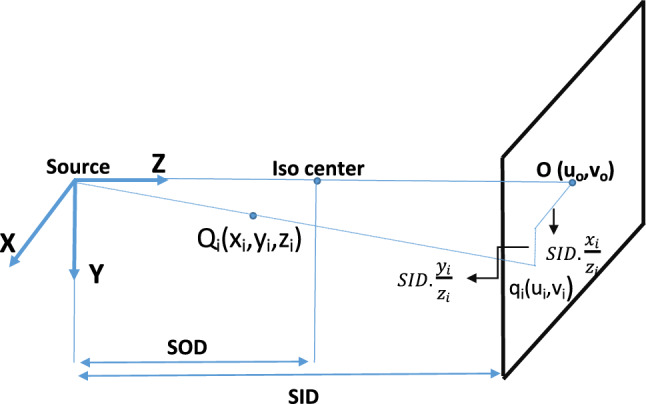
Geometry and mathematical model of angiographical projections [7, 8]

An X-ray imaging system, consisting of an X-ray source and a detector mounted on a C-arm, captures 2*D* projections of 3*D* structures. In biplane imaging, two C-arms provide dual views, enabling 3*D* reconstruction (Fig. 6). The projection of a 3*D* object point *Q*_*i*_ onto the image plane is *q*_*i*_(*u*_*i*_*,v*_*i*_) (Eq. 1, Fig. 7). *SID*_*i*_ and *SOD*_*i*_ refer to the source-to-image and source-to-object distances, respectively. The intrinsic matrix *K* includes pixel spacing (*pu*, *pv*), the skew parameter (*s*) to account for non-orthogonal axes, *SID*_*i*_, and principal point coordinates (*u*_0_, *v*_0_) (Eq. 2). Extrinsic parameters include rotation (*α*, *β* for LAO/RAO, left/right anterior oblique and CAU/CRA, caudal/cranial angulations) and translation (*T*) between views (Eq. 3–5). These parameters form the projection matrix (*P*), which maps 3D points to 2*D* image points (Eq. 3). Dual-view correspondence and triangulation are used to reconstruct 3*D* target object points [25, 26]. The mathematical framework and algorithms are detailed in prior works [7, 25]. The basic MATLAB functions are provided by the Visual Geometry Group, University of Oxford, under an open-source license [26].

1 $$ \left( {x_{i} ,y_{i} ,z_{i} } \right)^{T} \to \left( {{\text{SID}} \cdot \frac{{x_{i} }}{{z_{i} }} + u_{0} ,\;{\text{SID}} \cdot \frac{{y_{i} }}{{z_{i} }} + v_{0} } \right)^{T} = (u_{i} ,v_{i} )^{T} $$

2 $$ q_{i} = K\left[ {I\left| 0 \right.} \right]Q_{i} = PQ_{i} $$

$$K =\left[\begin{array}{ccc}\frac{{SID}_{j}}{pu}& \frac{{SID}_{j}}{pv}. s& {u}_{0}\\ 0& \frac{{SID}_{j}}{pv}& {v}_{0}\\ 0& 0& 1\end{array}\right]$$

3 $$ \begin{gathered} q_{{1}} = P_{{1}} X_{i} = K_{{1}} \left[ {I| \, 0} \right]Q_{i} \hfill \\ q_{{2}} = P_{{2}} X_{i} = K_{{2}} \left[ {R|T} \right]Q_{i} \hfill \\ \end{gathered} $$

4 $$ R = R_{x}( - \beta_{2})R_{y}(\alpha_{2}) \cdot (R_{x}( - \beta_{1})R_{y}(\alpha_{1}))^{- {1}} $$

5 $$ T = T_{{2}} - R \cdot T_{{1}} $$

### Iterative optimization

**Fig. 8:**
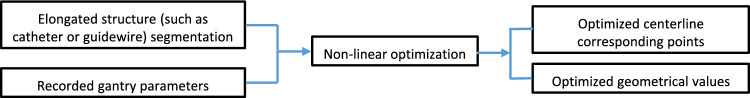
The whole self-calibration workflow

Based on predetermined criteria [28], the system’s geometrical parameters and correspondences were iteratively optimized using segmented catheter centerlines until the backprojection of the 3D reconstructed centerlines closely matched their real projections. The entire calibration workflow is shown in Fig. 8.

Intrinsic and extrinsic parameters were initialized with recorded system values and then optimized. Intrinsic matrix includes skew parameter, principal point coordinates (The location of the focal point of the C-arm within the X-ray image), and *SID*_*j*_ |_*j*=1*,*2_ which are initialized with 0, center of the image plane and source to detector distance, respectively. Extrinsic parameters, including the rotation matrix *R* (Eq.4) and translation vector *T* (Eq.5), were initialized using recorded gantry information based on Eq. 4 and 5.

The cost function (Eq. 6) comprises two errors: the Euclidean distance between the actual and back-projected catheter centerlines, and the directional vector differences between consecutive points in the actual and back-projected centerlines across both views.

6 $$\begin{aligned} &F\left( {SID_{j} ,s_{j} ,u_{oj}, v_{oj}, R,T}\right) \\&\quad = \sum\limits_{i = 1}^{n} \left[ {d(q_{1,i} -\widehat{{q_{1,i} }})^{2} + d(q_{2,i} - \widehat{{q_{2,i} }})^{2} }\right] \\&\quad + \sum\limits_{i = 1}^{n - 1} \left[ \left\| {\gamma_{1,i}- \widehat{{\gamma_{1,i} }}} \right\|^{2} - \left\| \gamma_{2,i} -\widehat{{\gamma_{2,i} }} \right\|^{2}  \right]\end{aligned}$$

The Levenberg–Marquardt algorithm [29], suitable for nonlinear least-square optimization, was used to minimize Eq. 6, with default MATLAB termination criteria [28] and parameter bounds.

### Corresponding points selection

Corresponding points and system parameters are iteratively optimized. From the centerline, 100 evenly spaced points in the first view were matched to corresponding points in the second view using epipolar lines. To enhance accuracy in finding corresponding points, 200 evenly spaced points were selected in the second view for denser potential matches [30].

**Fig. 9:**
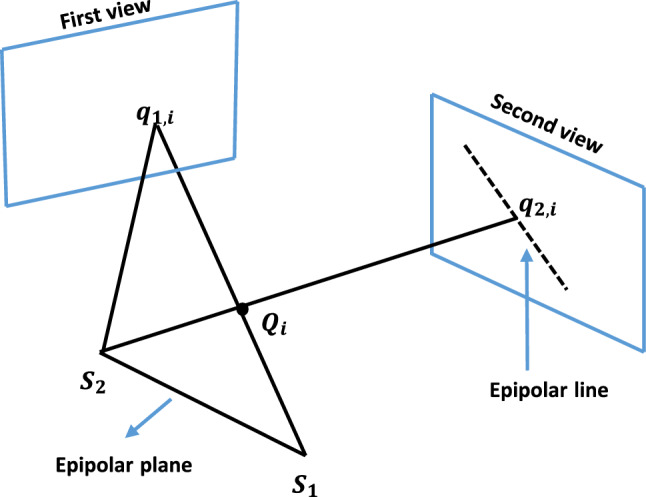
Epipolar relationship between two angiographic views [8]

For each point on the catheter centerline in the 1st view, the corresponding point in the 2nd view is chosen based on its proximity to the epipolar line (A limit of 2.25 pixels was experimentally set for corresponding points within a specific distance to the epipolar line (Fig. 9).) This threshold ensures accurate point matching, minimizing correspondence errors that are critical for reliable backprojection and system calibration. Without this limit, mismatches increase, significantly affecting calibration accuracy, especially in highly curved catheter cases like datasets 2 and 3, where backprojection errors rose to 1.9847 and 12.5359, respectively.

The intersection of the epipolar line with the catheter centerline or the closest point to it is selected as the corresponding point in the 2nd view. If multiple intersections exist, an additional criterion prevents sudden jumps by calculating two distances: (1) point-to-point distance between the previously computed and new corresponding points, and (2) distance along the catheter centerline. The difference between the two distances should be less than an experimental threshold of 1 pixel. This criterion was consistently satisfied across the three analyzed datasets; however, testing on more diverse cases with various catheter and guidewire appearances, varying levels of curvature, and different imaging views would further validate its robustness.

**Fig. 10:**
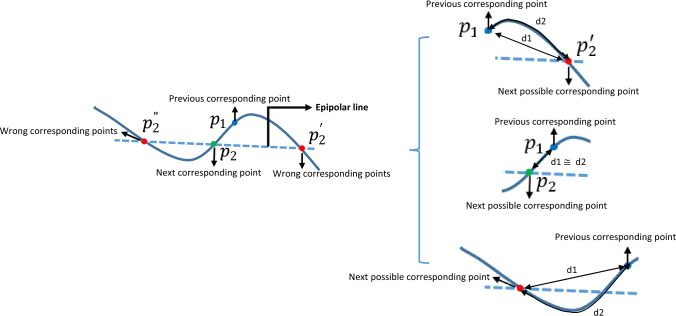
Corresponding point selection criteria, with three possible corresponding points

Additionally, a two-step optimization is used: first, catheter tips are treated as exact correspondences to optimize geometric parameters, which then initialize the optimization for the entire catheter, ensuring compliance with the aforementioned limitation criteria (Fig. 10).

## Experimental results

Our catheter segmentation method, detailed in Table 2, shows improved performance with hyperparameter optimization. The best results, based on Sensitivity, Specificity, Precision, FDR, and Accuracy, are highlighted, demonstrating superiority over other configurations.

**Table 2:** Performance comparison for different training configurations for optimizable ensemble classifier

Performance	Sensitivity	Specificity	Precision	FDR	Accuracy
Opt-Ens^1^ (1st frame); Mdl 1^2^	72.26	**99.43**	**86.41**	**13.59**	98.13
Opt-Ens (Multiple frames); Mdl 2	**96.68**	96.39	57.35	42.65	96.40

Values in bold font represent the best results

^1^Abbreviates for Optimizable Ensemble

^2^First trained model

**Fig. 11:**
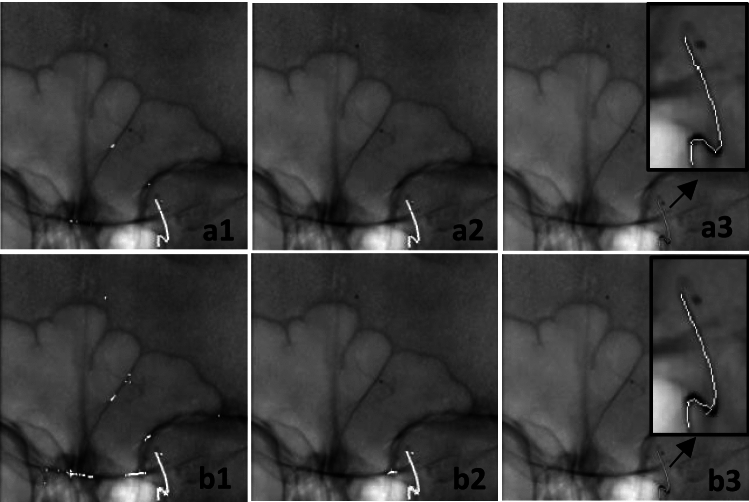
Segmentation result. (a1,b1). Initial segmented regions, (a2,b2). Segmented regions after FPs were removed considering vessel mask, (a3,b3). Catheter centerline

Figure 11 illustrates segmentation results using trained models. The first column shows segmented catheters, the second refines results by discarding false positives using the vessel map, and the third presents final centerlines (zoomed view) after manual corrections and interpolation to address disconnections.

**Fig. 12:**
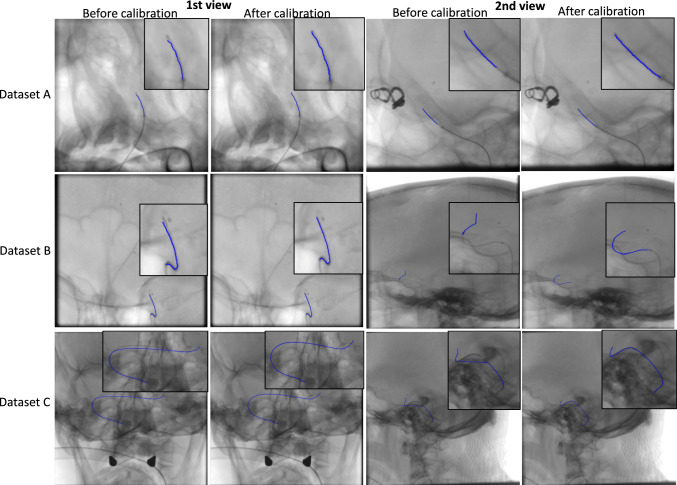
Backprojection of the catheter before and after self-calibration

Figure 12 shows catheter backprojection results (blue curves) before and after online calibration for three test sets in biplane views.

**Fig. 13:**
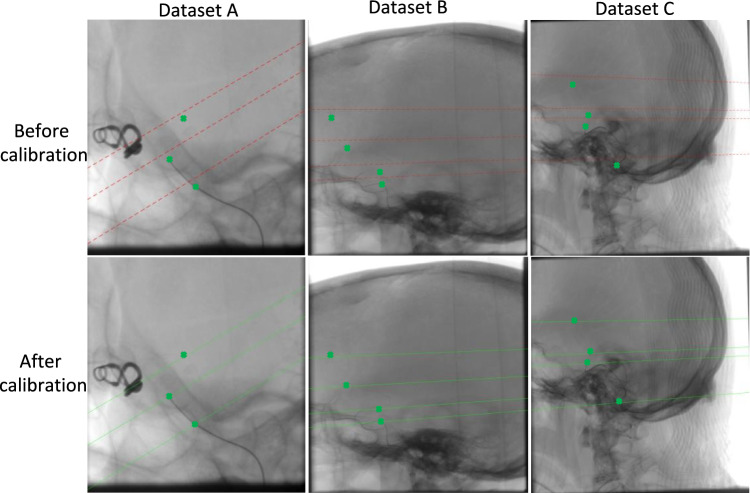
Epi polar lines before (red dotted line) and after calibration (green line) in the 2nd view for some specified points in the 1st view. Green stars demonstrate the exact correspondences in the 2nd view

Figure 13 shows how calibration affects the epipolar lines in the second view for three test sets. Corresponding epipolar lines for some specific points of the 1st view (catheter tip or the attached markers) are drawn in the second view. Red dotted line represents epipolar line before calibration while green line represents the epipolar line after calibration. Green stars show the exact corresponding points for the specified points of the 1st view.

Table 3 represents some geometrical parameters of the system (LAO/RAO angle, CAUD/CRAN angle, SID, and SOD for both projection views) before and after calibration.

**Table 3:** Geometrical system parameters before and after self-calibration

	Values (TS1)		Values (TS2)		Values (TS3)	
Parameters	BC^2^	AC^3^	BC	AC	BC	AC
α_1_^4^	− 22.90	− 22.70	− 5.90	− 5.88	0.30	− 2.70
α_2_^4^	− 103.80	103.97	− 93.20	− 93.20	− 90.10	− 87.10
β_1_^4^	− 27.60	− 27.74	− 1.40	− 4.40	− 0.70	− 3.47
β_2_^4^	13.40	12.65	0.20	− 1.60	− 0.50	− 0.64
SOD_1_^5^	716.44	718.58	745.97	745.41	745.99	748.99
SOD_2_^5^	750.00	747.95	749.99	750.56	750.00	747.00
SOD_1_^5^	1189	1187.49	1026	1026.43	1021.00	1018.00
SOD_2_^5^	1207.00	1208.48	1131.00	1130.70	1166	1168.99

^1^Test set

^2^Before calibration

^3^After calibration

^4^In degree

^5^In mm

The mean point to point Euclidean distance between the backprojection of the catheter and its real projection before and after calibration for both projection views for the current and previously proposed technique [7] are computed and shown in Table 4. To investigate the impact of offset variations on calibration accuracy, we evaluated the calibration performance under different offsets, including the source-todetector distance (SID) and source-to-object distance (SOD) for both the primary and secondary systems (both views). Table 5 presents the results for the tested offsets of 1 cm. Although the calibration converges for a 1 cm offset (results shown in Table 5), increasing the offset to 5 cm or 10 cm prevents the optimization from terminating, indicating that calibration is not feasible for larger deviations.

**Table 4:** A comparison between the current proposed method and previous method [7] using 2D backprojection error based on Euclidean distance for the catheter^1^ (mm)

2D error (mm)	Before calibration	Proposed method	Previous method
	1.28	0.01	0.11
	5.98	0.23	1.28
	5.08	0.22	0.36
Mean & std^2^	4.11 ± 2.61	0.15 ± 0.01	0.58 ± 0.50

^1^The catheter centerline obtained from the proposed segmentation technique was used both for calibration (currently proposed method) and computing error

^2^standard deviation

**Table 5:** Calibration Accuracy Under Varying Offset Levels—Variation of SID and SOD by 1 cm

	Original Data	SOD	SID
2D Back Projection Error (Euclidean Distance) [mm]	0.1193	0.1624	0.1939

To evaluate the performance of the proposed method, we conducted an experiment involving a microcatheter with two attached radio-opaque markers with an actual distance of 30 mm. The microcatheter was placed inside a vascular silicon phantom with an aneurysm-like structure, as shown in Fig. 1.

Four sets of angiograms were acquired, and the accuracy of the method was assessed based on the RMS error. The analysis revealed an average RMS error of 3.47% when compared to the true inter-marker distance of 30 mm.

## Discussion

This study evaluated a semi-automatic machine-learning approach for catheter detection and segmentation, combined with an online C-arm calibration technique utilizing known catheter lengths. The primary aim was to enhance catheter visualization in fluoroscopic sequences and improve the accuracy of 3D vascular reconstruction for interventional neuroradiology.

Our proposed method employed an optimizable ensemble classifier, trained on both the initial and multiple frames of sequences from the training dataset with optimized hyperparameters (see Table 2). This classifier outperformed other models in terms of specificity, precision, and false-discovery rate (FDR), achieving an accuracy of 98.13% and a sensitivity of 72.26%, while maintaining low false positives. Although a second model achieved higher sensitivity, it resulted in increased false positives, which compromised its performance in specificity, precision, and FDR. Low number of FPs highlights the effectiveness of our method in handling low signal-to-noise ratio images with overlapping structures.

In this study, we utilized a catheter with a defined length, such as the distance between two radio-opaque markers or from the tip to a visible marker, which is particularly advantageous for intracranial endovascular interventions where such catheters with radio-opaque markers are commonly used.

Computed catheter centerlines, combined with gantry-recorded data, were fed into a nonlinear optimization algorithm to iteratively refine the system’s geometrical parameters by minimizing the cost function (Eq. 6). The results demonstrated that leveraging catheter centerlines for C-arm calibration improved 3D reconstruction accuracy. Specifically, the average backprojection error was reduced from 4*.*11 ± 2*.*61 before calibration to 0*.*15 ± 0*.*01 after calibration, highlighting a substantial enhancement in precision (Table 4)).

Figure 12 illustrates the backprojection results for three test sets before and after calibration in biplane views. While significant deviations from the real catheter projection were observed before calibration, the backprojection closely aligned with the real projection after calibration, underscoring the effectiveness of the proposed method. Furthermore, the average error for our current technique was notably lower compared to previous approaches, as detailed in Table 4. To validate the method in 3D space, we employed a microcatheter with two radio-opaque markers spaced 30 mm apart, placed within a vascular silicon phantom featuring aneurysm-like structures (Fig. 1). This setup replicated designs from prior studies that used an IVUS catheter with a fixed length of 30 mm and intracoronary guidewires with eight markers spaced 15 mm apart [10, 24]. Our method achieved an average RMS error of 3.47% in estimating the inter-marker distance, further confirming its accuracy and reliability confirming the accuracy of our approach in real-world clinical scenarios. Compared to previous calibration methods that often relied on anatomical bifurcation points, our approach using catheter centerlines is particularly applicable in cases where bifurcations were unavailable or difficult to detect.

Guidewire visualization in low-dose fluoroscopy presents significant challenges due to reduced image quality and overlapping anatomical structures. Our method detects and segments catheter, which eventually improves navigation while minimizing radiation exposure. A key strength of our approach lies in the use of handcrafted features, such as those derived from the Frangi filter. These features capture domain-specific characteristics that aid in detecting vessel-like structures, complementing machine learning models and contributing to overall performance improvements. By integrating interpretable features, our method ensures reliability and enhances decision-making in complex imaging scenarios. While deep learning techniques like U-Net have demonstrated success in segmentation, their reliance on large datasets increases the risk of overfitting, particularly in medical imaging where annotated data is often limited. By combining machine learning with handcrafted features, our approach addresses this limitation, maintaining high accuracy without excessive data requirements. This balance between interpretability and performance makes our method a robust and practical solution for real-world applications.

Despite the promising results, certain limitations must be addressed. The requirement for manual segmentation correction and instrument length specification introduces variability and may limit clinical adoption.

In this study, we employed a wavelet-based approach to extract candidate regions and incorporated handcrafted features, such as the Frangi vesselness filter, to enhance interpretability and capture vessel-like structures similar to catheters. While we did not directly compare our method to CNN-based models, we acknowledge their success in detection tasks due to their ability to learn hierarchical features. However, handcrafted features offer advantages in terms of interpretability, computational efficiency, and domain-specific relevance, particularly when training data is limited. Although our machine learning approach effectively reduced false positives, achieving higher sensitivity remains challenging due to the low contrast and overlapping structures in fluoroscopic images. Future work could explore hybrid models that integrate handcrafted and CNN-based features to leverage the strengths of both. Such combinations may improve detection accuracy and generalizability by capturing both explicit structural cues and learned representations. Additionally, expanding the dataset with more diverse fluoroscopic sequences could enhance model robustness and reduce reliance on user input.

The ability to accurately segment catheters and refine C-arm calibration in real time has significant implications for interventional neuroradiology. Enhanced catheter visibility can improve procedural safety by reducing reliance on high-dose fluoroscopy, while more precise calibration facilitates better 3D vascular reconstructions for aneurysm treatment planning and complex interventions. By addressing current limitations and incorporating automated enhancements, this technique has the potential to improve fluoroscopy-based guidance in neurointerventional procedures.

## Conclusion

This study presents an online calibration technique for curved interventional instruments, such as catheters with adhered markers, and a correspondence selection approach for conditions with multiple intersections, potentially improving 3D vascular reconstruction accuracy. A novel machine learning technique for catheter segmentation is also proposed. Future work could evaluate this method on diverse C-arm datasets for generalizability and develop a fully automatic version. This approach can also be applied to other endovascular procedures involving elongated instruments with radio-opaque markers.
